# Overexpression of *MdATG18a* in apple improves resistance to *Diplocarpon mali* infection by enhancing antioxidant activity and salicylic acid levels

**DOI:** 10.1038/s41438-018-0059-5

**Published:** 2018-11-01

**Authors:** Xun Sun, Liuqing Huo, Xin Jia, Runmin Che, Xiaoqing Gong, Ping Wang, Fengwang Ma

**Affiliations:** State Key Laboratory of Crop Stress Biology for Arid Areas/Shaanxi Key Laboratory of Apple, College of Horticulture, Northwest A&F University, 712100 Yangling, Shaanxi China

## Abstract

Marssonina apple blotch, caused by *Diplocarpon mali*, is one of the most serious diseases of apple. Autophagy plays a key role in pathogen resistance. We previously showed that MdATG18a has a positive influence on drought tolerance. Herein, we describe how overexpression (OE) of *MdATG18a* enhances resistance to *D. mali* infection, probably because less H_2_O_2_ but more salicylic acid (SA) is accumulated in the leaves of OE apple plants. Expression of *chitinase*, *β-1,3-glucanase*, and SA-related marker genes was induced more strongly by *D. mali* in OE lines. Transcript levels of other important *MdATG* genes were also drastically increased by *D. mali* in OE plants, which indicated increased autophagy activities. Taken together, these results demonstrate that OE of *MdATG18a* enhances resistance to infection by *D. mali* and plays positive roles in H_2_O_2_-scavenging and SA accumulations. Our findings provide important information for designing strategies which could induce autophagy to minimize the impact of this disease on apple production.

## Improving resistance to apple blotch disease

Apple plants that express high levels of a gene involved in the destruction of damaged and redundant cell components demonstrate improved resistance to a serious fungal infection. Fengwang Ma and colleagues of China’s Northwest A&F University in Shaanxi examined the effect of overexpressing the gene *MdATG18a* on *Diplocarpon mali* fungal infection in apple plants. This gene was previously found to promote drought tolerance in apple plants and is known for its involvement in autophagy, an immune response to infection that removes damaged cell organelles. The researchers found that plants overexpressing *MdATG18a* were more resistant to developing Marssonina apple blotch disease compared to wild apple plants, most likely due to improvements in autophagy activity. The team concludes that strategies designed to induce autophagy could improve apple resistance to fungal infection.

## Introduction

Marssonina apple blotch is one of the most serious apple diseases, caused by the fungus *Diplocarpon mali*^1^. This pathogen infects apple plants using both necrotrophic and biotrophic strategies, suggesting that it behaves in a hemibiotrophic manner^2^.

*Diplocarpon mali* ascopores released from overwintered apothecia are responsible for primary infections and conidia produced in acervuli are considered the inoculum for secondary infections^2^. Upper surface of infected leaves show greyish brown leaf spots and small black acervuli before leaves become yellow and fall off^2^. Visible symptoms appear in the second half of June and spreads at a faster rate during July–August with average temperature between 23.5 and 25.4 °C and frequent rains of moderate to high intensity^1^. The development of this disease is positively correlated with relative humidity and rainfall. *Diplocarpon mali* also produce pseudo-conidia on overwintered disease leaves^3^, which are dispersed by rain splashes and mainly infect old leaves near the bottom of shoots from the beginning of apple leaf growth^3^. Ascospores mature from mid May to the end of June, and are discharged in response to rain and dispersed with wind^3^. Infection leads to premature defoliation and affects fruit quality and quantity^4^.

Because of long-term interactions with pathogens, plants have developed sophisticated defense systems that can inhibit or alleviate the harm caused by pathogens. When infection occurs, the innate immunity-pathogen-associated molecular pattern (PAMP)-triggered immunity (PTI) is generally activated^5,6^. However, PTI can be overcomed by a secondary immunity-effector triggered immunity^5,6^. These two phases of plant immunity can be induced by several defense reactions, including oxidative bursts and hormones. Generation of H_2_O_2_, an example of a stable reactive oxygen species (ROS), is considered a universal plant response to pathogen attack^7^. Main ROS-scavenging mechanisms include enzymatic and non-enzymatic antioxidants, both of which regulate redox homeostasis in plant cells. Pathogenesis-related (PR) proteins can be induced by plant basal defense responses. For example, chitinase and *β*-1,3-glucanase may have direct antimicrobial activities due to their roles in the hydrolyzation of respective chitin and glucans in the fungal cell wall^8^.

Plant hormones are involved in growth, development, reproduction, and stress reponse. For example, salicylic acid (SA) is a crucial component in disease resistance signaling^9^. The SA-related pathway is activated by biotrophic pathogens^10^. SA also activates phenylalanine ammonia-lyase and polyphenol oxidase, which are involved in phenolic compound synthesis and cell wall strengthening^11,12^. Upregulation of *PR* genes often indicates the induction of plant defense. In some crops, SA application enhances expression of some *PR* genes to confer pathogen resistance^13,14^. Transcripts of *PR1* and *PR5* are coupled with the accumulation of endogenous SA, which makes both genes molecular markers for the SA-dependent signaling pathway^15,16^.

Autophagy, a conserved cellular process in eukaryotes, is important for recycling nutrients and cytoplasmic components^17–20^. In yeast, WD40 repeat-containing protein AuTophGy-related (Atg) 18 is able to bind phosphatidylinositol 3-phosphate (PtdIns(3)P) and phosphatidylinositol 3,5-bisphosphate (PtdIns(3,5)P_2_)^21^. The PtdIns(3)P binding capacity of Atg18 is required for recruitment of Atg8 and Atg16 during phagophore formation^22^. The access of Atg4 to Atg8-phosphatidylethanolamine at phagophore assembly site is disturbed by the Atg18/21 complex to prevent a premature cleavage. However, the Atg18/21 complex dissociates and allows Atg4 to cleave Atg8-PE and release Atg8 after completion of the autophagosome. Therefore, a key aspect of post-translational regulation of autophagy by Atg4 is closely related with Atg18/21^22^. Autophagy has both pro-survival and pro-death roles in regulating hypersensitive response programmed cell death (HR-PCD) and plant immunity system under biotic stresses^23–26^, depending on factors such as plant age and pathogens. Autophagosome formation in *Arabidopsis* autophagy-deficient mutant *atg18a* was disturbed^27^, resulting in greater susceptibility to necrotrophic fungal pathogens in cooperation with jasmonate-mediated and WRKY33 (containing a highly conserved heptapeptide motif WRKYGQK 33)-mediated signaling pathways^26^. However, *Arabidopsis atg5*, *atg10*, and *atg18a-2* plants show enhanced resistance to *Pseudomonas syringae* pv. tomato^28^. Liu et al.^29^ have demonstrated that *Nicotiana tabacum* with *ATG6/BECLIN1* silenced showed increased cell death under viral infection. Some regulatory factors also function in plant immunity by regulating autophagy. RabG3b, a GTP (guanosine triphosphate)-binding protein, facilitates HR-PCD by enhancing autophagosome formation under avirulent bacterial pathogens infection in *Arabidopsis*, thereby having a positive effect in immunity^30^. Thus, the molecular mechanisms and functions of autophagy are implicated in the complex life and death decisions under different pathological situations in plants^25^.

As one of the most serious apple diseases in Asian regions, Marssonina apple blotch causes great losses in fruit production. The function of autophagy in response to the infection by the pathogen is largely unknown. Two transgenic apple lines expressing *MdATG18a* under the control of the CaMV35S promoter demonstrated that the apple autophagy-related gene *MdATG18a* enhances plant resistance to drought stress via an improved ROS-scavenging system and activating autophagy^31^. In addition, these two lines also showed enhanced tolerance to nitrogen depletion^32^. *MdATG18a* transcription level was greatly increased by *D. mali* infection and overexpression (OE) of the gene was associated with improved resistance to *D. mali* in apple plants, probably due to lower ROS accumulations, higher accumulations of SA, and regulation on some *PR* genes because of improved autophagy activities. These findings demonstrate that MdATG18a plays a key role in the resistance of apple plants to *D. mali*.

## Results

### Pathogen-responsive expression patterns of *MdATG18a* to *D. mali* infection

**Fig. 1:**
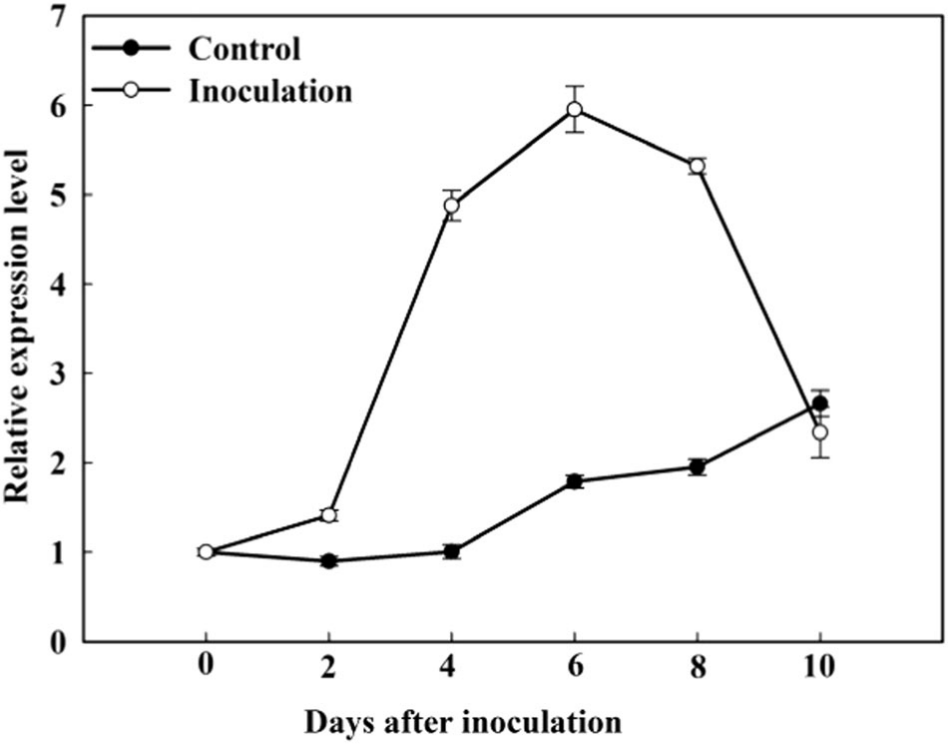
Changes in the expression of *MdATG18a* after *D. mali* infection in WT apple plants. Expression levels were calculated relative to the expression of *Malus EF-1α* mRNA. Expression of *MdATG18a* at 0 dpi was set to “1”. Data are means of three replicates with SD. Experimental data were presented as means ± SD

Expression of *MdATG18a* can be induced by universal stresses^33^. To examine whether this is also true in response to pathogen attacks, we monitored the transcription level of this gene upon pathogen infection in wild-type (WT) apple leaves and found that the expression was up-regulated in the leaves of the control (mock-inoculated) at the end of the treatment period. The transcripts of the inoculated leaves peaked at 6 days post-inoculation (dpi) (more than 4-fold higher over 0 dpi) before gradually decreased over time (Fig. 1), indicating that *MdATG18a* was responsive to *D. mali* infection in apple.

### *MdATG18a* OE enhanced resistance to *D. mali* infection

**Fig. 2:**
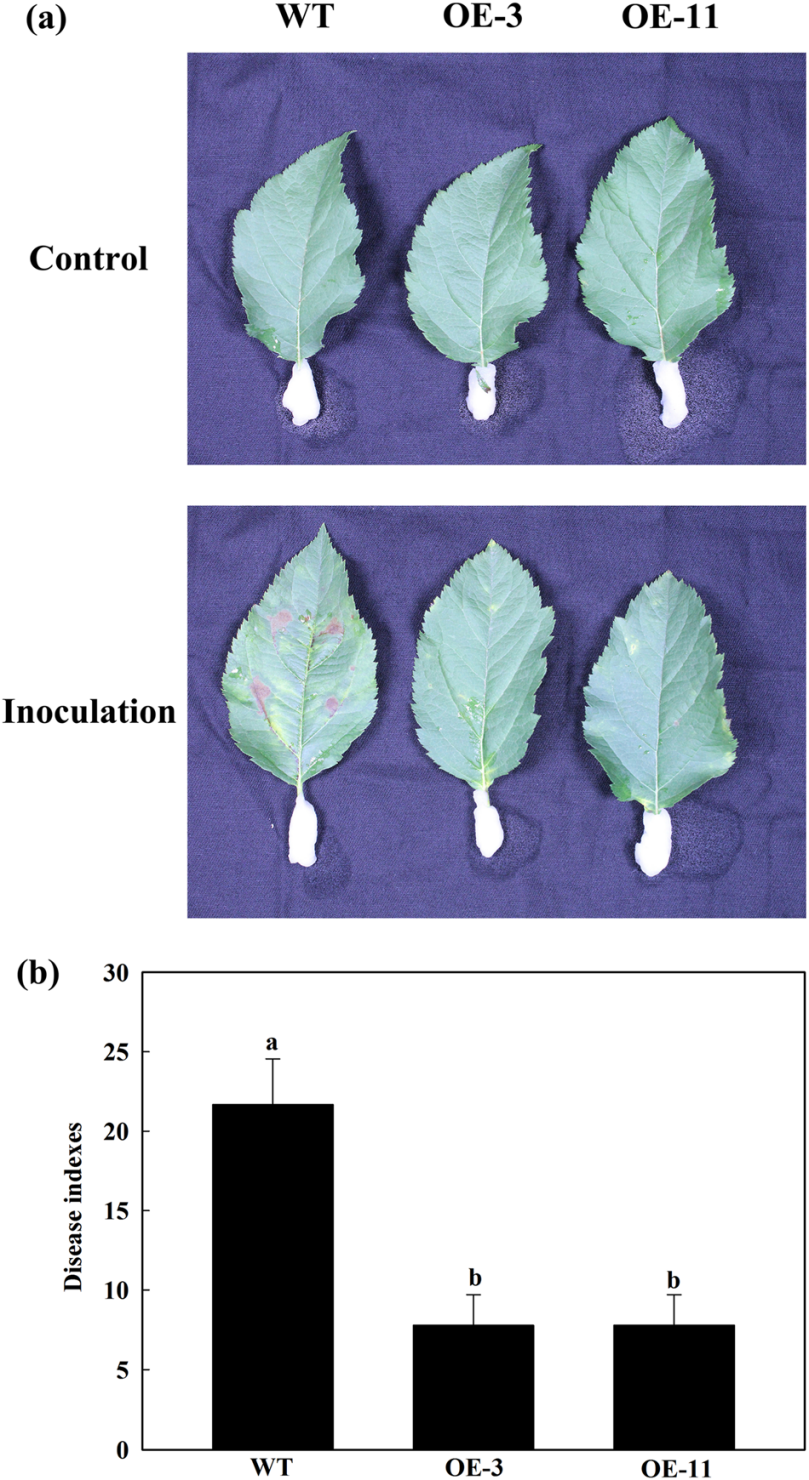
*MdATG18a* overexpression enhanced resistance to *D. mali* infection in transgenic apple plants. **a** Phenotypes of WT and OE lines at 10 dpi. **b** Disease indices of WT and OE lines at 10 dpi. Data are means of three replicates with SD. Experimental data were presented as means ± SD. Different letters indicate significant differences between treatments, according to one-way ANOVA Tukey’s multiple range tests (*P* < 0.05)

We used two transgenic lines of *MdATG18a* OE apple which were obtained previously for further investigation of possible functions of MdATG18a in pathogen resistance. Excised, mature leaves from apple plants were inoculated with conidial suspensions of *D. mali*. As shown in Fig. 2a, the inoculation resulted in serious necrotic spots and chlorosis in WT but failed to cause extensive tissue damage in OE plants. Disease indices on WT leaves were more than 2.5-fold that of OE leaves (Fig. 2b).

### *MdATG18a* OE resulted in reduced H_2_O_2_ accumulation and increased antioxidant activity and AsA-GSH cycling upon *D. mali* infection

**Fig. 3:**
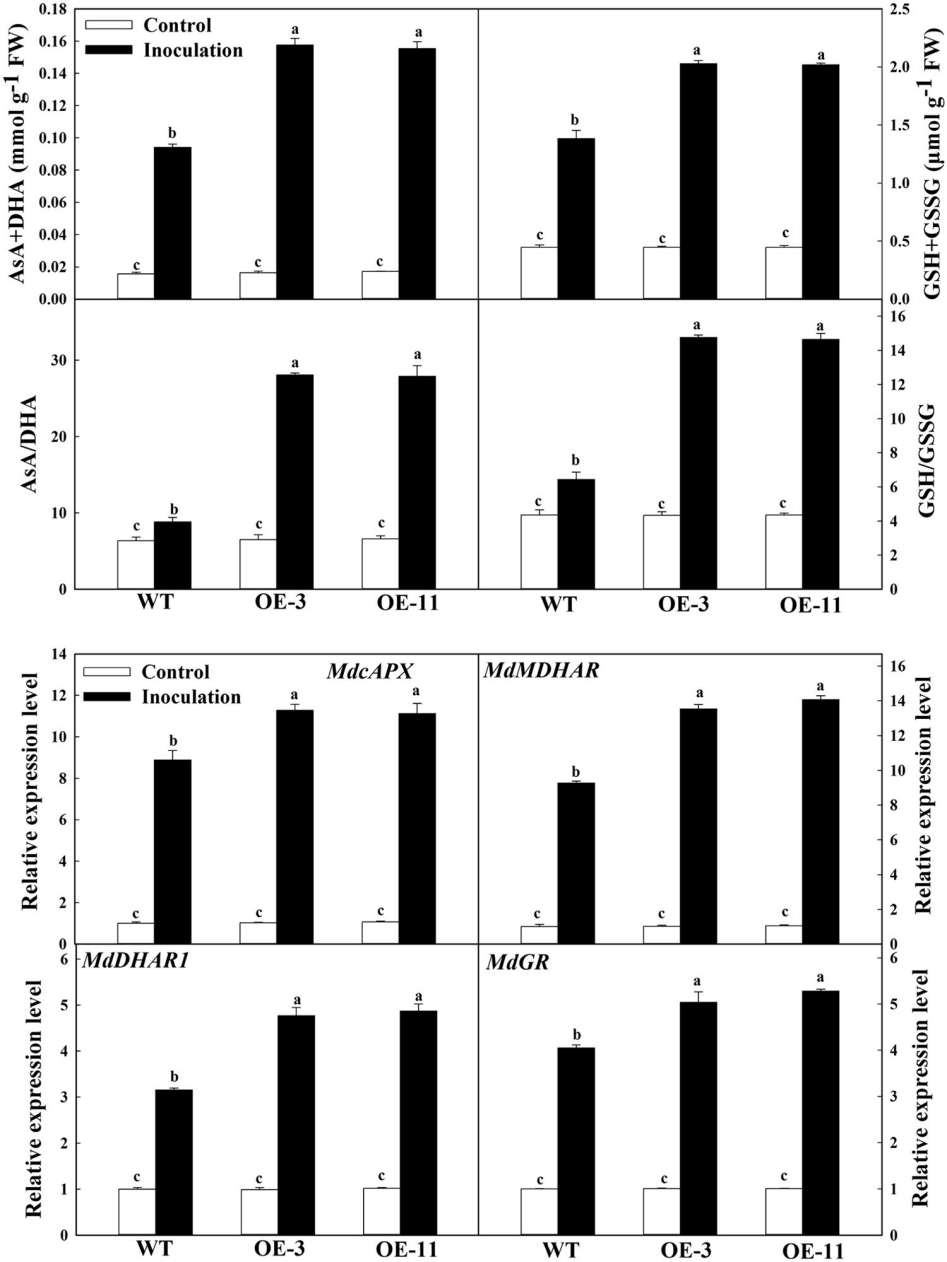
*MdATG18a* overexpression resulted in reduced H_2_O_2_ accumulation and increased antioxidant activity and AsA-GSH cycling upon *D. mali* infection. **a** H_2_O_2_ concentrations and H_2_O_2_-scavenging enzymes activity. **b** Concentrations of antioxidants and transcript levels for genes involved in AsA-GSH cycle. H_2_O_2_-scavenging enzymes and concentrations of antioxidants were measured at 10 dpi. Expression levels were calculated relative to the expression of *Malus EF-1α* mRNA at 6 dpi. Expression in WT under control conditions was set to “1”. Data are means of three replicates with SD. Experimental data were presented as means ± SD. Different letters indicate significant differences between treatments, according to one-way ANOVA Tukey’s multiple range tests (*P* < 0.05)

To analyze the redox status after leaf inoculation with *D. mali*, we measured the concentrations of H_2_O_2_ and activities of two major H_2_O_2_-scavenging antioxidant enzymes. H_2_O_2_ increased significantly in all infected leaves, but the level of H_2_O_2_ was significantly lower in OE leaves than in WT leaves at 10 dpi (Fig. 3a). Enzyme activities were increased in response to elevated H_2_O_2_ accumulations, whereas under control conditions, the levels of catalase (CAT) and peroxidase (POD) did no differ significantly between WT and OE plants. After infection, CAT activity in the OE lines was increased by 1.5 times over that in the WT. A similar pattern was observed for the POD activity.

As an antioxidant system, the ascorbic acid (AsA)-GSH cycle is involved in scavenging H_2_O_2_ under stress. We evaluated the status of ascorbate and glutathione to determine the changes in *MdATG18a* expression in the AsA-GSH cycle (Fig. 3b). No significant changes in the levels of total ascorbate and total glutathione were found between the OE lines and WT plants under the control conditions. However, the levels of total ascorbates and ratio of AsA to dehydroascorbic acid (DHA) were significantly higher in the transgenic plants than in the WT plants. Similarly, the levels of total glutathione pool and the reduced glutathione/oxidized glutathione (GSH/GSSG) ratio was also determined. We further examined the transcript levels for major genes in the AsA-GSH cycle. Under the normal conditions, expression of *Malus* × *domestica cytoplasm ascorbate peroxidase* (*MdcAPX*), *Malus* × *domestica monodehydroascorbate reductase* (*MdMDHAR*), *Malus* × *domestica dehydroascorbate reductase 1* (*MdDHAR1*), and *Malus* × *domestica glutathione reductase* (*MdGR*) did not differ significantly among genotypes. However, at 6 dpi, the transcript levels of these four genes were strongly increased, especially in the transgenic lines. For example, expression of *MdcAPX* was 1.27 times higher in OE samples than in WT (Fig. 3b).

### *MdATG18a* OE enhanced SA accumulation and increased the expression of SA-related *PR* genes upon *D. mali* infection

**Fig. 4:**
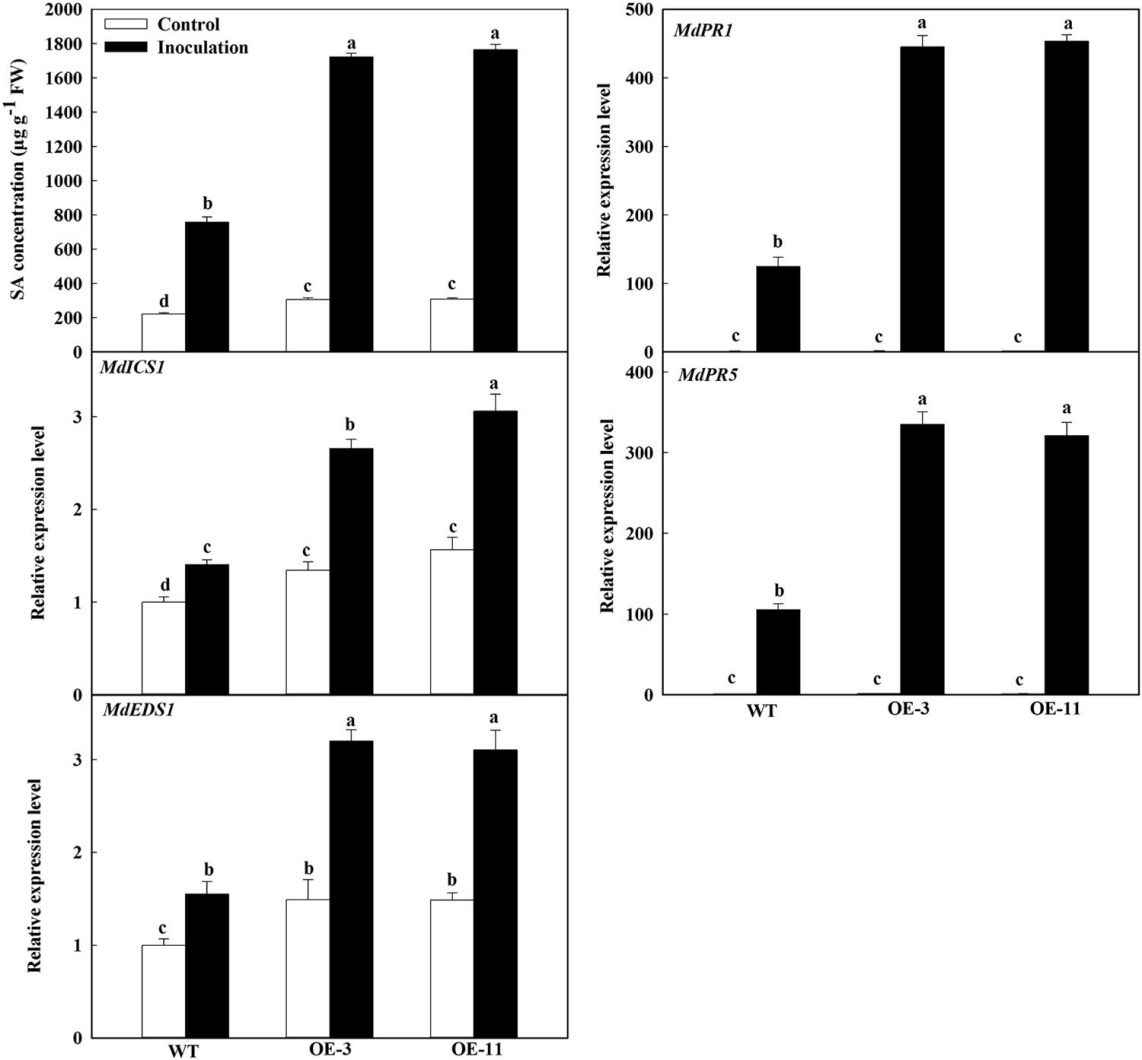
*MdATG18a* overexpression induced SA synthesis and increased the expression of SA-related *PR* genes. SA content was measured at 10 dpi. Expression of *MdICS1*, *MdEDS1*, *MdPR1*, and *MdPR5* was analyzed at 6 dpi. Expression levels were calculated relative to the expression of *Malus EF-1α* mRNA. Expression in WT under control conditions was set to “1”. Data are means of three replicates with SD. Experimental data were presented as means ± SD. Different letters indicate significant differences between treatments, according to one-way ANOVA Tukey’s multiple range tests (*P* < 0.05)

It has been reported that SA signaling is implicated in the autophagy-related immunity response^34,35^. To examine the relationship between SA and MdATG18a upon *D. mali* infection, we analyzed SA concentrations and the expression of two SA biosynthesis genes. For the mock control and inoculation, the SA level was significantly higher in the OE lines than in the WT (Fig. 4). For example, in response to inoculation, the level of SA in the OE plants was more than 2-fold that of the WT. Expression of *Malus* × *domestica isochorismate synthase 1* (*MdICS1*) and *Malus* × *domestica enhanced disease susceptibility 1* (*MdEDS1*) was up-regulated in parallel with the change in SA concentrations. Furthermore, their expression was up-regulated in the OE plants even under the mock control conditions, and their induction by *D. mali* infection was much stronger than in the WT. Similarly, the expression level of SA-related *PR* genes, *MdPR1* and *MdPR5*, was significantly higher in the transgenic lines than in the WT at 6 dpi on the inoculated leaves (Fig. 4).

### *MdATG18a* OE promoted the activity and expression of chitinase and β-1,3-glucanase upon *D. mali* infection

**Fig. 5:**
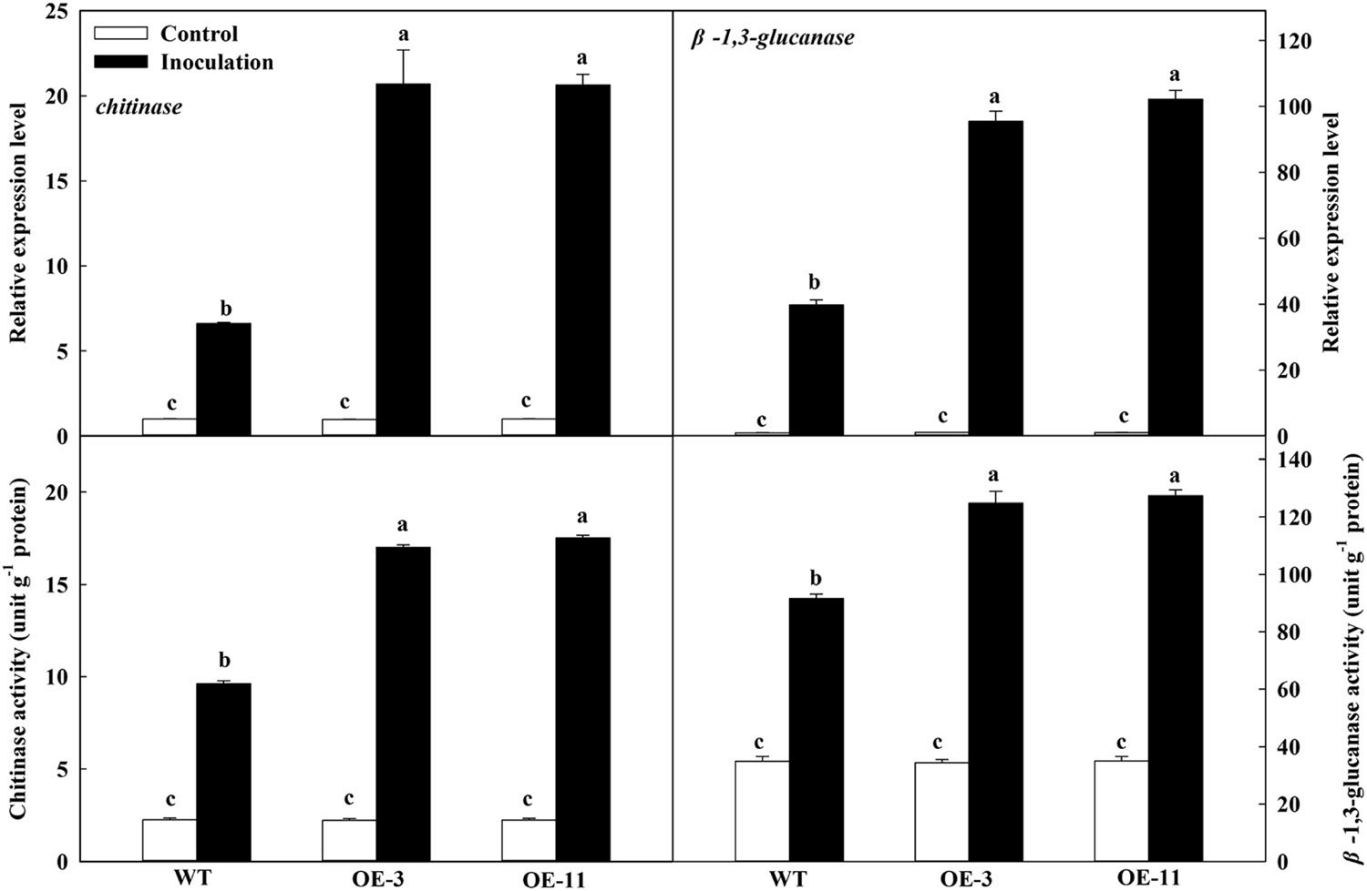
*MdATG18a* overexpression elevated chitinase and β-1,3-glucanase activity upon *D. mali* infection. Expression was analyzed at 6 dpi. Chitinase and β-1,3-glucanase activity was measured at 10 dpi. Expression levels were calculated relative to the expression of *Malus EF-1α* mRNA. Expression in WT under control conditions was set to “1”. Data are means of three replicates with SD. Experimental data were presented as means ± SD. Different letters indicate significant differences between treatments, according to one-way ANOVA Tukey’s multiple range tests (*P* < 0.05)

In the mock inoculation control, expression of *chitinase* and *β-1,3-glucanase* did not differ significantly among genotypes, but was greatly up-regulated after inoculation; the transcript levels were more than two times higher in the OE plants than in WT at 6 dpi (Fig. 5). Upon inoculation, the chitinase and β-1,3-glucanase activities were significantly greater in the OE lines than in the WT.

### *MdATG18a* OE increased the expression of other *MdATG*s upon *D. mali* infection

**Fig. 6:**
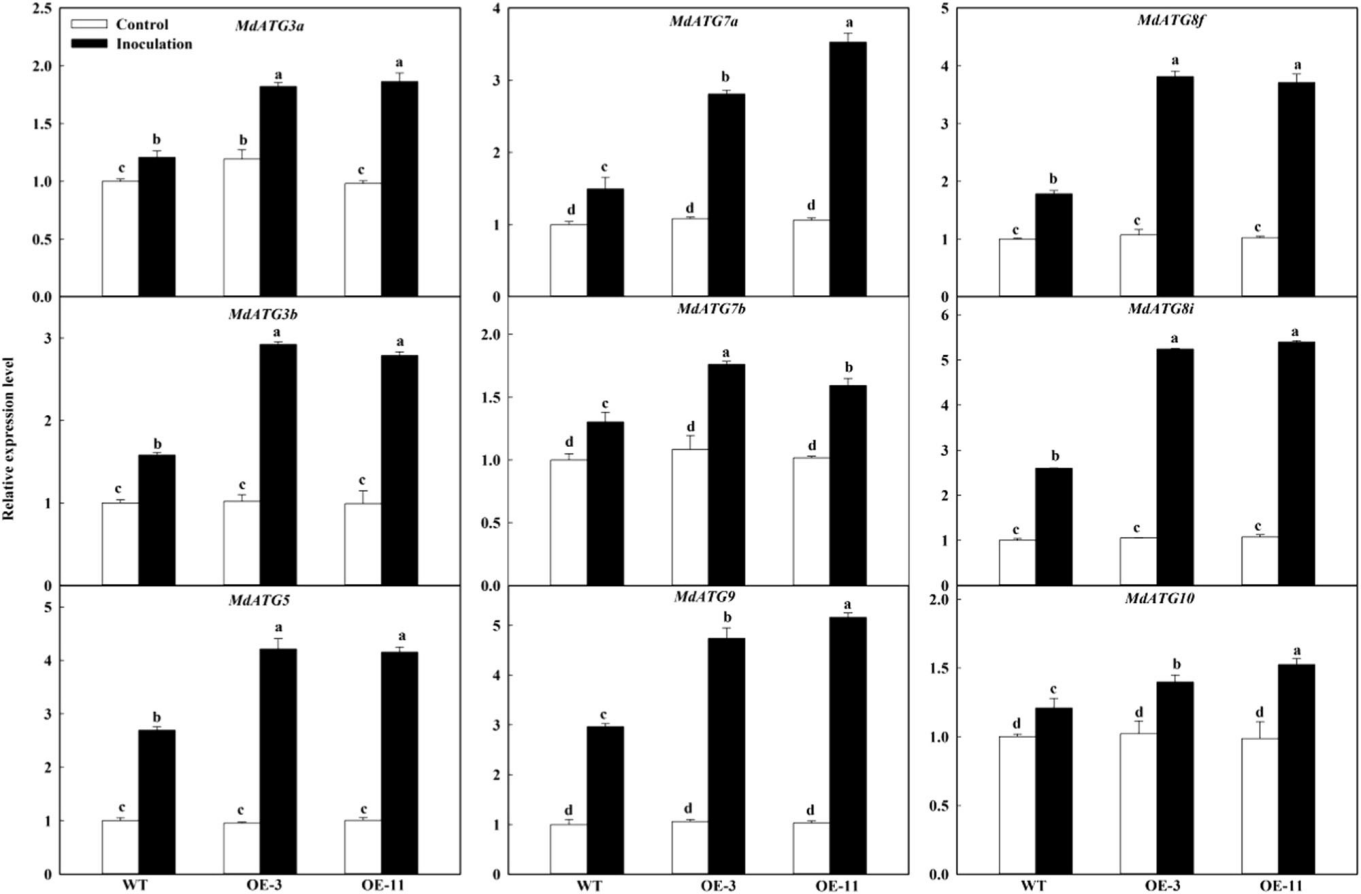
Changes in transcription level of apple autophagy-related genes under infection. Total RNA was isolated after 6 dpi, and expression levels were calculated relative to the expression of *Malus EF-1α* mRNA. Expression in WT under control conditions was set to “1”. Data are means of three replicates with SD. Experimental data were presented as means ± SD. Different letters indicate significant differences between treatments, according to one-way ANOVA Tukey’s multiple range tests (*P* < 0.05)

To examine the occurrence of autophagy under pathogen infection, we analyzed expression patterns of several important *ATG* genes. Under the mock control conditions, expression of *MdATG3a*, *MdATG3b*, *MdATG5*, *MdATG7a*, *MdATG7b*, *MdATG8f*, *MdATG8i*, *MdATG9*, and *MdATG10* did not differ among genotypes (Fig. 6). However, at 6 dpi, the expression of all studied genes was significantly higher in the OE lines than in WT. These results suggested that the transcripts of other important *ATG* genes were more responsive to *D. mali* infection in the OE leaves than in the WT, which might lead to stronger autophagy induction to build defense immunity.

## Discussion

Autophagy is thought to participate in the immune response to infection by various pathogens^25,26,34–36^. We previously demonstrated that *MdATG18a* is induced by various stress factors in WT apple plants^33^. The expression of autophagy-related genes and the autophagosome formation are also up-regulated in *Arabidopsis* in response to *Botrytis cinerea* infection^26^. Here, we explored the function of MdATG18a in apple in response to infection by *D. mali*. Expression of *MdATG18a* was up-regulated by inoculation with *D. mali* and its OE led to increased resistance against the pathogen, which might have resulted from increased ROS-scavenging capacity and SA synthesis. In the inoculated tissues, transcripts of *chitinase*, *β-1,3-glucanase*, *MdPR1*, *MdPR5*, and other important *MdATG*s were significantly up-regulated in the OE plants than in the WT. These results suggested that OE of *MdATG18a* enhances resistance to infection by *D. mali*.

Our previous work showed that *MdATG18a* transcripts were strongly induced under drought, heat and oxidative stresses, and peaked before a gradual decline^33^, suggesting the feedback regulation of *MdATG18a* under stress. The highly expressed *MdATG18a* appears to be indispensable to the stress response in the late stage of stress. The present work showed that expression of *MdATG18a* also peaked at 6 dpi and followed by a gradual decline in response to infection by *D. mali* (Fig. 1). We also found increased expression of *MdATG18a* even though in the mock-inoculated control (Fig. 1), probably because the mock-inoculated control (detached leaves under dark condition) may also induce *MdATG18a* expression^33^.

Pathogens rely upon several toxic factors, including stimulated ROS, to promote host cell death. H_2_O_2_ has some possible functions in the host response to pathogen infection and is the most versatile and stable ROS^37^. It also coordinates a localized HR when attacked by pathogen^7^. Autophagy is believed to be involved in degrading oxidized proteins and regulating ROS levels under various stresses^31,38–40^. Autophagy induced by pathogens ensures cell survival by preventing excess ROS production, removing excess and damaged organelles, and regulating organelles to a normal level that fulfills cellular requirements^25^. Antioxidant activity might contribute to infection by *D. mali*^41,42^. We previously reported that OE of *MdATG18a* in apple reduces ROS accumulations by promoting the antioxidant system under the drought stress^31^. Herein, we found that less H_2_O_2_ was accumulated in the OE lines under inoculation. This probably resulted from increased CAT and POD activities as well as improved AsA-GSH cycling.

As PR proteins, chitinase and *β*-1,3-glucanase can help cells resist pathogen infection by decomposing the pathogen cell walls^8^, and the products from decomposed cell walls can then activate a series of defense reactions^43^. Expression of *chitinase* and *β-1,3-glucanase* can be up-regulated by pathogen infection^42^. For example, transgenic lines of *Vitis vinifera* that contain chitinase (from *Triticum aestivum*) and *β*-1,3-glucanase have higher transcripts of *chitinase* and *β-1,3-glucanase* and show enhanced resistance to *Plasmopara viticola*^44^. After inoculation with *D. mali*, transcript levels of *β-1,3-glucanase* in apple are higher in resistant plants of *M. sieversii* against *D. mali* than in susceptible plants of *M. prunifolia*^41^. Similarly, our results indicated that *MdATG18a* OE up-regulated the expression of *chitinase* and *β-1,3-glucanase* and improved the activities of these two enzymes post-inoculation. This suggests that chitinase and β-1,3-glucanase are involved in limiting the extent of pathogen invasion and colonization in the OE plants.

The ATG18a protein interacts with WRKY33 and plays a key role in resisting to necrotrophic pathogens in *Arabidopsis*^26^. A small increase in the bacterial growth was observed in *ATG6*-silenced *Arabidopsis* plants over WT only at the early stages of *PstDC3000* (*Pseudomonas syringae* pv. tomato *DC3000*) infection^36^. The same bacterium also increased its growth in *Arabidopsis atg7* mutants^45^. All of these findings suggest that autophagy plays a positive role in resisting pathogen. However, Wang et al.^35^ found both enhanced resistance to powdery mildew and mildew-induced cell death in an *Arabidopsis atg2* mutant^35^. The *atg18a-2* mutant has also shown mildew-induced cell death similar to the *atg2* mutant and enhanced resistance to the powdery mildew (*Golovinomyces cichoracearum*)^46^. These reports demonstrated that autophagy has a negative role in resisting pathogens. These contrasting reports about the role of autophagy in response to pathogen infection are probably related to SA signaling and the age of the leaves that were studied^25,34^. Young leaves contain less SA and reduced cell death than older leaves, regardless whether they are WT or mutants, suggesting young leaves are more susceptible to *Pst-AvrRpm1* than older leaves. In mature leaves, which accumulate higher levels of SA than in young leaves, pathogen-induced cell death is suppressed in WT plants but not in *atg* mutants^25,34^. SA is involved in both local defense responses at the infection site and the activation of systemic resistance^6,10,47^. SA is essential for early senescence and immunity-related PCD in autophagy-deficient mutants^34^. Our OE lines contained more SA under both the inoculation and control conditions. Two SA synthesis genes, *MdICS1* and *MdEDS1*, were more strongly induced in the OE lines after inoculation. This seems to contradict previous findings that *atg* mutants accumulate more SA^35,46^. We previously demonstrated that ectopic expression of apple *MdATG8i*, *MdATG7*, and *MdATG3* in *Arabidopsis* slightly promoted leaf senescence and bolting^48–50^. In the present work, the SA level was slightly higher in the OE lines than in the WT under mock control conditions. After inoculation, SA synthesis was up-regulated, especially in the OE lines. This greater accumulation of SA might have contributed to the enhanced resistance to pathogen infection. We speculate that this response resulted from the activation of autophagy via *MdATG18a* OE, based on our finding that major *MdATG* genes were significantly up-regulated in transgenic plants. To investigate why OE of *MdATG18a* also enhanced SA biosynthesis, a knock-out of *MdATG18a* or interference of *MdATG18a* in apple would be one viable strategy to pursue. *MdATG18a* knock-out/interference apple plants may be similar to *Arabidopsis atg18a* mutant that may have increased SA level compared with WT plants. In the SA signaling defense pathway, the enhanced plant resistance to biotrophic and hemibiotrophic pathogens results from up-regulated *PR* genes^51^. Likewise, we noted here that *MdPR1* and *MdPR5* were much more induced by *D. mali* in the OE lines than in the WT, demonstrating that regulation of the SA pathway is required for pathogen resistance.

In summary, we have functionally characterized *MdATG18a* under pathogen infection. Transgenic apple plants had enhanced resistance to *D. mali*, possibly because of a more-reductive redox state and a higher concentration of SA in the OE plants. Expression levels of *chitinase*, *β-1,3-glucanase*, and SA-related genes were greatly up-regulated by *D. mali* in the OE lines. This might be explained by the improvements in autophagy activity, as supported by increased transcription of important *MdATG*s. These results provide further insight about the function of autophagy in apple disease resistance. We can design strategies which could induce autophagy to curb the impact of future infections according to our results that increased autophagic activity could increase resistance of apple to pathogen.

## Materials and methods

### Treatment and plant materials

Tissue-cultured plants of *Malus* × *domestica* cv. Royal Gala were cultivated as described previously^31^. OE transgenic and WT plantlets were transferred to small plastic pots (8.5 × 8.5 × 7.5 cm^3^) containing a mixture of loam/perlite (1:1, v:v). After 1 month of adaptation in a growth chamber, one plant was moved to each large plastic pot (30 × 26 × 22 cm^3^) filled with a mixture of clay/sand/organic substrate (5:1:1, v:v:v), and grown in the greenhouse. They were watered regularly and supplied with half-strength Hoagland’s nutrient solution (pH 6.0) once a week. We used theses plants for examining their resistance to *D. mali* infection after 90 days under such conditions.

For inoculation and disease evaluation, the ninth to twelfth healthy apple leaves from the base of a stem (fully mature leaves) were excised from WT and OE plants. The inoculation assay was performed as previouly described^52^. A total of 120 WT leaves in two plastic boxes were used for *MdATG18a* expression following *D.mali* infection. Three or four entire WT leaves were sampled for per repeat at each 0, 2, 4, 6, 8, and 10 dpi. Leaves were sampled thrice as three repeats with total 10 leaves at each sampling time. Total 60 leaves in two plastic boxes were inoculated from 20 WT/OE-3/OE-11 plants, with four inoculation sites per leaf. Three or four entire leaves were sampled for per repeat at 0, 6, and 10 dpi. Leaves were sampled thrice as three repeats with total 10 leaves at each sampling time. The plastic boxes were sprayed with sterile water twice a day to keep humid conditions for 10 days^52^.

### Inoculum preparation

A monospore culture of *D. mali* originated from diseased apple leaves showing Marssonina blotch symptoms which were collected from a research orchard at the College of Horticulture, Northwest A&F University, Yangling (34°20′N, 108°24′E), Shaanxi Province, China. Single spores were isolated by a method reported previously^53,54^. The inoculum suspension was prepared and adjusted to 1 × 10^6^ conidia per milliliter as previously described^52,55,56^.

### Evaluation of infection degree

At 10 dpi, disease severity for each leaf was scored on a scale of 0 to 5, where 0 = no disease symptoms, while 1 = 1 to 10%, 2 = 11 to 30%, 3 = 31 to 50%, 4 = 50 to 80%, or 5 = 80 to 100% of the entire leaf area showed lesions^41,52^. The following formula was used:

$${\mathrm{Disease}}\,{\mathrm{index}} = \frac{{{\mathrm{Sum}}\,{\mathrm{of(severity}}\,{\mathrm{score \times the}}\,{\mathrm{number}}\,{\mathrm{of}}\,{\mathrm{leaves}}\,{\mathrm{in}}\,{\mathrm{that}}\,{\mathrm{severity}}\,{\mathrm{score) \times 100}}}}{{{\mathrm{Total}}\,{\mathrm{number}}\,{\mathrm{of}}\,{\mathrm{leaves}}\,{\mathrm{ \times 5}}}}.$$

These experiments were conducted three times.

### RNA extraction and real-time PCR

Total RNA was extracted according to a CTAB method^57^. First-strand cDNA synthesis and real-time PCR were performed as previously described^31^. *Malus* elongation factor 1α gene (*EF-1α*; DQ341381) were used as internal control. Primer sequences for real-time PCR are listed in Table S1. Each experiment was repeated three times biologically, based on three separate RNA extracts from three repeats.

### Extraction and assay of H_2_O_2_ and antioxidant metabolites

Apple leaves (0.1 g) were used for quantifying H_2_O_2_ and antioxidant metabolites. Activities of CAT and POD were determined according to established protocols^39^. Five percent (w/v) of trichloroacetic acid was used for H_2_O_2_ extraction and quantification as described previously^58^. Six percent (v/v) HClO_4_ was used for AsA and DHA extraction, while 5% (v/v) sulfosalicylic acid was used for GSH and GSSG extraction. Concentrations of AsA, DHA, GSH, and GSSG were quantified as described previously^59^.

### Extraction and assays of chitinase and β-1,3-glucanase activity

Extraction and assays of chitinase and β-1,3-glucanase activity were performed as described previously^42,60–63^.

### Quantification of SA

SA was extracted and quantified as described previously^64,65^. Leaf samples (0.1 g) were homogenized in liquid nitrogen and placed in 1 mL of 90% methanol. 3-Hydroxybenzoic acid (Sigma) was added as internal standard. Extracts were analyzed though a fluorescence detector (excitation at 305 nm and emission at 405 nm) on a ZORBAX SB-C18 column (Agilent Technologies, Santa Clara, CA, USA).

### Statistical analysis

Experimental data are presented means ± standard deviations of three independent replicates. Data were analyzed via analysis of variance (ANOVA), and mean values were compared by Tukey’s multiple range test (*p* < 0.05). All the statistical analyses were performed using SPSS18 statistical software package (IBM SPSS Statistics, Chicago, IL, USA).

## Electronic supplementary material

Table S1
