# Supplementary material for: Overexpression of MdATG18a in apple improves resistance to Diplocarpon mali infection by enhancing antioxidant activity and salicylic acid levels
Source: Hortic Res. 2018 Nov 1;5:57. doi: 10.1038/s41438-018-0059-5 (PMC6210185; doi:10.1038/s41438-018-0059-5)
Supplement: Supplementary file 1 — Table S1 [file 41438_2018_59_MOESM1_ESM.docx]

| **Name** | **Sequence (5**'**-3**'**)** | **Purpose** |
| --- | --- | --- |
| q*ATG18a* | F: ATGATTCCAGGCTTGCCTGCTTTG | Quantitative expression of *MdATG18a* |
|  | R: TGCAGCAAAGTTCCGTCGAGAGTA |  |
| *EF-1α* | F: ATTCAAGTATGCCTGGGTGC | Real-time PCR using *Malus* *EF-1α* as reference gene |
|  | R: CAGTCAGCCTGTGATGTTCC |  |
| q*MdICS1* | F:GCTTTACGCCCAAGACCA | Quantitative expression of *MdICS1* |
|  | R:CGGCAACGCTGACCAACT |  |
| q*MdEDS1* | F: TGGAGAAAGTGATTTTGGAGAAGC | Quantitative expression of *MdEDS1* |
|  | R: AGAACCAGATTGTGACAAACGC |  |
| *Chitinase* | F: TGGAGGATGGGAAAGTGC | Quantitative expression of *Chitinase* |
|  | R: GGGTGAGTTGGATGGGTC |  |
| β-1, 3-glucanase | F: TGCCGTAGGAAACGAAAT | Quantitative expression of *β-1, 3-glucanase* |
|  | R: TGATGGAGGAAAGGAATT |  |
| q*MdPR1* | F: GCAGCAGTAGGCGTTGGTCCCT | Quantitative expression of *MdPR1* |
|  | R: CCAGTGCTCATGGCAAGGTTTT |  |
| q*MdPR5* | F: AACTAGCATCCAAAGCTAGCC | Quantitative expression of *MdPR5* |
|  | R: CCACAGTCTGCAGTTTCACAAG |  |
| q*ATG3a* | F: AAGGGGGCGGAGATGGTTC | Quantitative expression of *MdATG3a* |
|  | R: GCACTTAGAGACGAGGTTATCGC |  |
| *qATG3b* | F: AGGGAGATGGTTTTGAAACAGA | Quantitative expression of *MdATG3b* |
|  | R: ACTTAGAGACGAGGTTATCGC |  |
| q*ATG5* | F: GCAGGTCGTGTTCCAGTTC | Quantitative expression of *MdATG5* |
|  | R: CCTCCTCCTCCTTGTATCTCAA |  |
| q*ATG7a* | F: GCGGATATGAGCAACCTTGGC | Quantitative expression of *MdATG7a* |
|  | R: ATCAATAGGCGCAACGACATCA |  |
| q*ATG7b* | F: ATCGGTAACAGGAGTAAGTCGG | Quantitative expression of *MdATG7b* |
|  | R: TTTATCAAGCGCATGAAAGCCT |  |
| q*ATG8f* | F: TCGTAGACAATGTCCTCCCAGC | Quantitative expression of *MdATG8f* |
|  | R: CCAAATGTGTTCTCGCCACTGT |  |
| q*ATG8i* | F: GCAGCAGGCTTCACTTGACTCC | Quantitative expression of *MdATG8i* |
|  | R: GGAATCCATGCGACTGGCTGTT |  |
| q*ATG9* | F: ACTTCATGCGTCAGCCTTCAGA | Quantitative expression of *MdATG9* |
|  | R: CGTTCCTCCAATCCAACCGTTG |  |
| q*ATG10* | F: TGGAACCAGCGAGTGGATGAAG | Quantitative expression of *MdATG10* |
|  | R: ACAACTGAGAGCCAAGACACCA |  |
| q*cAPX* | F: AACTACAAGGGATGAAGCC | Quantitative expression of *MdcAPX* |
|  | R: CAACGAGGATGATAACCAG |  |
| q*MDHAR* | F: CCATACTTCTATTCCCGCTCCT | Quantitative expression of *MdMDHAR* |
|  | R: CGACCACCTTCCCGTCTTT |  |
| q*DHAR1* | F: AGTGGACGGTTCCAGCAGA | Quantitative expression of *MdDHAR1* |
|  | R: AGTGGACGGTTCCAGCAGA |  |
| q*GR* | F: GTTCAGCGACAAGGCGTAT | Quantitative expression of *MdGR* |
|  | R: TCAACCGATTTCCATTTCC |  |
